# Organic Fertilizers Promote Accumulation of Mineral Nutrients in Citrus Leaves by Affecting Soil Biochemical Properties and Bacteria

**DOI:** 10.3390/plants14182826

**Published:** 2025-09-10

**Authors:** Lei Yang, Min Wang, Jianjun Yu, Shuang Li, Lin Hong

**Affiliations:** Fruit Tree Research Institute, Chongqing Academy of Agricultural Sciences, Chongqing 401329, China; leir8512@126.com (L.Y.); wm950918@126.com (M.W.); jerksion@163.com (J.Y.); sclishuang61@163.com (S.L.)

**Keywords:** ‘*Orah*’, organic fertilizer, mineral element, soil enzyme, soil bacteria

## Abstract

This study aimed to investigate the influence of different organic fertilizers and their concentrations on the growth of ‘*Orah*’ (*Citrus reticulata* Blanco) seedlings, as well as on the mineral nutrient contents, chemical and biological properties, and microbial community of the soil. Five types of organic fertilizers and three concentrations were studied. The seedling growth indexes, leaf mineral elements, soil mineral elements, soil enzyme activity, and soil microorganisms were measured. The results showed that organic fertilization significantly increased the contents of eight mineral elements in leaves, depending on the types and concentrations used. Specifically, rapeseed cake fertilizer was found to significantly increase the content of iron (Fe), manganese (Mn), and zinc (Zn) in the leaves. Furthermore, compared with applying only chemical fertilizers or no fertilizers at all, the application of organic fertilizer significantly increased the content of soil organic matter (SOM) and several mineral elements in the soil. The bacterial species composition of soil treated with common organic fertilizer and bio-organic fertilizer, and sheep manure were similar; however, the bacterial composition was significantly different in the soil which been treated with rapeseed cake compared to these other three fertilizers. Additionally, PICRUSt function predicting indicates that the core microbial community in the rapeseed cake group could promote synthesis and the transport of sugar, iron and other substances. Organic fertilizer can change soil chemical and biological properties by affecting the core microbial community structure, and further promote accumulation of mineral elements in the leaves of citrus seedlings.

## 1. Introduction

The scale of citrus cultivation and its yield in China are the largest in the world, contributing significantly to increasing farmers’ income and promoting economic development [1,2,3]. It is an important industry supporting rural revitalization [4]. In China, citrus is usually grown in mountainous areas, but poor soil affecting plant growth is a serious problem [5]. Fruit farmers in these producing areas have always used fertilization to improve tree growth, fruit quality, and production. However, the excessive use of chemical fertilizers, the insufficient supply of organic fertilizer and improper application methods lead to a serious shortage of soil organic matter [6]. The long-term and extensive application of chemical fertilizers has brought about a series of environmental and industrial problems: On the one hand, long-term application of chemical fertilizers has destroyed the structure and function of the original microbial communities, reducing the effectiveness of soil nutrients [7,8,9]; on the other hand, the application of chemical fertilizers accelerates the acidification process in soil [10,11], and then activates soil heavy metals [12,13]. This seriously affects the growth and fruit quality of citrus and impedes the sustainable development of the citrus industry in China. In contrast, the rational and scientific use of organic fertilizers plays an important role in improving soil quality and promoting plant growth.

Organic fertilizers are mainly composed of animal and plant residues (such as livestock manure and crop straw) obtained after a harmless treatment and decomposed organic materials. They have the characteristics of comprehensive nutrient balance, lasting fertilizer effect, contain many functional bacteria and microorganisms, and have been used to fertilize the soil, repair soil damage, and improve soil [14,15,16]. A lot of theoretical research and practice shows that organic fertilizers can improve physicochemical properties, microbial community structure, and the fertility level of soil [17]. Furthermore, they can suppress the activity of soil pathogenic microorganisms, reduce the incidence of plant diseases, and increase soil microbial diversity [18]. Organic fertilizers also play an active role in improving soil physicochemical properties in order to produce agricultural products of a high quality [19]. However, most existing studies focus primarily on the short-term effects of organic fertilizer application on soil physicochemical properties, biological characteristics, and crop quality [20], or on the remediation of soils affected by continuous cropping obstacles and the control of soil-borne diseases [21], while the impact of fertilizer application on citrus growth and soil vitality has not been systematically researched [22].

In order to improve fruit yield and quality, an increasing number of fruit farmers have recognized the advantages of utilizing organic fertilizers. Currently, there are many kinds of organic fertilizers available from various sources. The types of organic fertilizers commonly used in production include ordinary commercial organic fertilizers, bio-organic fertilizers with active microorganisms added, manure, residue left by various plant seeds after oil extraction (cake fertilizer), and organic fertilizer mixed with a certain proportion of inorganic fertilizer (organic–inorganic complex fertilizers). Organic materials such as farmyard manure, cakes of plant origin, vermicompost, and microbial bio-fertilizers are important components of the bio-organic concept of citrus cultivation [23]. Sheep manure enables Khasi Mandarin to achieve its maximum plant height, plant length and crown volume, whereas vermicompost, pig manure, poultry manure and neem cake cannot [24]. Rapeseed cake fertilizer can effectively enhance the quality and flavor of crops [25]. However, there are huge price differences between different types of organic fertilizers, and the nutrient characteristics and prices of these fertilizers differ widely in the market, which makes it difficult for fruit farmers to choose the best one. Therefore, it is essential to scientifically and systematically analyze how different types and concentrations of organic fertilizer can improve the soil environment in orchards and affect the growth of citrus trees and fruit quality. In this study, we selected the five most commonly used types of base fertilizers in Chongqing to investigate their effects on the growth of ‘*Orah*’ (*Citrus reticulata* Blanco) seedlings, leaf mineral nutrition and soil microecology. Furthermore, we provide a theoretical basis and technical support for the rational use of organic fertilizers in citrus planting.

## 2. Materials and Methods

### 2.1. Site Description

We conducted our study in the Xianfeng experimental base of the Chongqing Agricultural Science College (116°34′ E, 36°50′ N). The study area, characterized by mineral purple soil, is located in a warm, temperate, subhumid monsoon climate zone, with an annual average temperature of 17.4 °C. The annual effective accumulated temperature above 10 °C ranged from 5500 °C to 6500 °C, with annual precipitation and evaporation totals of 930 mm, annual evaporation of 2095 mm, and a frost-free period of 206 days.

### 2.2. Experimental Design

This experiment was generally designed with an organic fertilizer application group and a non-application group. The organic fertilizer application group was treated with varying concentrations of four organic materials: organic–inorganic fertilizer, common organic fertilizer (A), bio-organic fertilizer (B), sheep manure (C) and rapeseed cake (D). The non-organic fertilizer application group included four treatments currently used in production: broadcasting compound fertilizer (CK1), broadcasting urea (CK2), irrigating with urea solution (CK3), and no fertilizer application (CK4). The organic–inorganic-blend fertilizer and common organic fertilizer were purchased from Chongqing Liangping Fengjiang Biotechnology Co., Ltd. (Chongqing, China). The bio-organic treatment was purchased from Chongqing Wanzhi Biotechnology Co., Ltd. (Chongqing, China). The sheep manure, rapeseed cake, compound fertilizer and urea were purchased from Chongqing Yunyang Yihuo Agricultural Materials Co., Ltd. (Chongqing, China). These five types of fertilizers were separately and thoroughly blended with the soil from common citrus orchards at mass concentrations of 1%, 5%, and 10%. Subsequently, 20 kg of each of the resulting mixtures was transferred into seedling pots at the same time, with 9 replicate pots for each treatment. To ensure the normal growth of the young trees, a 5% urea solution was applied at 15-day intervals across all five organic fertilizer treatments. In addition, CK1 and CK2 consisted of monthly applications of 25 g compound fertilizer or urea alone. For CK3, only 5 ‰ urea was applied twice per month, and CK4 was not fertilized. This experiment was designed based on practical production, and considered to more effectively highlight the effects of different organic fertilizers on the growth and on soil conditions of young citrus trees. The specific experimental treatments are detailed in Table 1. The basic nutrient conditions of various fertilizers and citrus orchard soil are shown in Table 2. The citrus materials used in this experiment were one-year-old ‘*Orah*’ (*Citrus reticulata* Blanco) seedlings grafted onto trifoliate orange (*Poncirus trifoliata* L. Raf) rootstock. The citrus seedlings were about 60 cm in height and 6 mm in stem diameter. After thoroughly mixing the fertilizer with the soil, uniformly-sized citrus seedlings were planted in the pots. Seedlings with consistent tree vigor were selected for potted planting, with every 3 seedlings forming one biological replicate, resulting in a total of 3 biological replicates. The seedlings were treated with a unified standard. After one year of cultivation, the relevant biological and chemical properties of the seedling plants and the soil were determined.

### 2.3. Data Processing and Analysis

#### 2.3.1. Biomass, Leaf and Soil Chemical Index Determination

Stem diameter, plant height and the number of terminal shoots of the last section are the most representative indicators of the growth of citrus seedlings. Therefore, we adopted these three indicators to represent the influence of fertilizers on plant growth. In addition, we also determined the contents of 10 mineral elements in the citrus leaves, including nitrogen (N), phosphorus (P), potassium (K), iron (Fe), calcium (Ca), magnesium (Mg), zinc (Zn), manganese (Mn), copper (Cu) and boron (B). A total of 11 soil chemical and 4 soil enzymes’ activities were determined, including soil organic matter (SOM), total nitrogen (TN), available phosphorus (AP), available potassium (AK), iron (Fe), calcium (Ca), magnesium (Mg), zinc (Zn), manganese (Mn), copper (Cu), boron (B), soil urease, soil sucrase, soil dehydrogenase and acid phosphatase. The TN and AN contents were calculated using the Kjeldahl method. The Olsen method was used to measure the amount of AP. After NH4OAc extraction, the amount of AK in the soil was measured using a flame photometer. The contents of Ca, Mg, Fe, Mn, Cu, Zn and B were determined by the HNO_3_-HClO_4_ digestion method [26,27]. The soil enzyme activity levels were determined using kits (Suzhou Grace Biotechnology Co., Ltd. (Suzhou, China)) [28,29]. The citrus leaf mineral elements, soil organic matter, mineral elements and enzyme activities of soil were determined by Amida Biotechnology Co., Ltd. Chongqing, China, and each index was measured with three biological replicates.

#### 2.3.2. The Quantification of 16S rRNA Genes

The 16S rRNA genes were quantified externally by Genesky Biotechnologies Inc. (Shanghai, China). The 16S rDNA V3-V4 region was amplified using a polymerase chain reaction (PCR) (95 °C for 2 min, followed by 27 cycles at 98 °C for 10 s, 62 °C for 30 s, and 68 °C for 30 s, and a final extension at 68 °C for 10 min) using the primers 341F (5′-CCTACGGGNGGCWGCAG-3′) and 805R (5′ GACTACHVGGGTATCTAATCC 3′), where the barcode was an eight-base sequence unique for each sample. Multiple spike-ins with identical conserved regions for 16S rRNA genes and variable regions replaced by random sequences with 40% GC content were artificially synthesized, and an appropriate mixture with known gradient copy numbers of spike-ins was then added to the sample DNA. An MiSeq sequencer was used to amplify and sequence the V4-V5 region and spike-ins of the 16S rRNA gene to generate at least 10 million 2 × 250-bp paired-end raw reads (Genesky, Shanghai, China). The main instruments and equipment used include the NanoDrop 2000 (10x Genomics, Thermo Fisher Scientific, Waltham, MA, USA), the Invitrogen Qubit 3.0 Spectrophotometer (Thermo Fisher Scientific, Waltham, MA, USA), Agilent 2100 Bioanalyzer (Agilent Technologies, Santa Clara, CA, USA), Illumina MiSeq Benchtop Sequencer (Illumina, San Diego, CA, USA), the ABI 2720 Thermal Cycler (Thermo Fisher Scientific, Waltham, MA, USA), and the Eppendorf 5810R Centrifuge (Eppendorf, Hamburg, Germany).

#### 2.3.3. Illumina MiSeq Sequence Processing and Function Analysis

TrimGalore (v0.4.5; Felix Krueger, Berlin, Germany) and Mothur (v1.39.5 [https://github.com/mothur/mothur/releases/tag/v1.39.5 (accessed on 20 May 2023)]) were used to remove the adaptor and primer sequences, respectively. At the opposite end, the reads were merged and filtered, and the remaining reads were then clustered into operational taxonomic units (OTUs) with a sequence similarity level of at least 97%. The OTUs were annotated, and a standard curve was established between the read count and the number of incorporated DNA copies. The absolute copy number of the bacterial OTUs was calculated using the read count of the corresponding bacterial OTUs. Bacterial community richness and diversity were assessed with the ACE and Shannon indices using the UPARSE pipeline. Similarity between the bacterial communities was estimated by principal component analysis (PCA) using the Bray-Curtis measure. A bacterial interaction network (using Pearson correlations) was produced using the OmicStudio tools (https://www.omicstudio.cn/tool, (accessed on 12 May 2024)), and the network map was made using Gephi 0.9.4. Raw sequencing data were deposited in the Genome Sequence Archive (https://ngdc.cncb.ac.cn/gsa/, (accessed on 9 August 2023)) (Accession Number CRA011447). Redundancy analysis (RDA) was performed by R using the vegan packages. Functional profiles of the microbial communities in biofilm samples were predicted using PICRUSt (phylogenetic investigation of communities by reconstruction of unobserved states).

#### 2.3.4. Statistical Analysis

Totals of 5925, 5922, 5733, 4543, and 4890 OTUs were related to the bacteria used for treatments A, B, C, D, and CK. The keystone species (genera) were identified based on the betweenness centrality score. The biomass, leaf and soil properties under the different treatments were compared using one-way analysis of variance (ANOVA) in IBM SPSS Statistics (version 20; SPSS Inc., Somers, NY, USA). In the analysis of variance, each combination of different fertilizer types and concentrations that supported the normal growth of young citrus trees was considered as a distinct treatment for statistical analysis. Significance was determined using Tukey’s test (*p* < 0.05 or *p* < 0.01), and the confidence level was 95%.

## 3. Results

### 3.1. Influence of Different Organic Fertilizer Treatments on Citrus Growth and Leaf Nutrients

#### 3.1.1. Influence of Different Organic Fertilizer Treatments on Citrus Seedling Growth

The different types and concentrations of fertilizers affected the growth of the ‘*Orah*’ seedlings in significantly varied ways. After a one-month application period, all the seedlings exhibited wilting and mortality when an organic–inorganic compound fertilizer was used as the base fertilizer, indicating its unsuitability for this purpose. Subsequently, a one-way analysis of variance (ANOVA) was conducted for each of the 19 combinations of fertilizer and concentration, with each combination treated as an individual experimental condition. As shown in Table 3, the common organic fertilizer, bio-organic fertilizer, sheep manure, and rapeseed cake significantly promoted the growth of the citrus seedlings. Among these fertilizers, a fertilizer concentration of 10 g/kg resulted in the largest stem diameter, plant height and the number of final branches. However, higher concentrations of the same fertilizers were found to inhibit growth. Even the control groups that received only chemical fertilizers or no fertilizers at all exhibited the significant effect on the growth of young trees; however, on average, the control groups grew the least compared to those given the organic fertilizers. Overall, the citrus seedlings treated with 10 g/kg organic fertilizer exhibited a superior growth performance compared to those treated with 100 g/kg rapeseed cake.

#### 3.1.2. Effects of Different Organic Fertilizer Treatments on Mineral Elements in Citrus Leaves

The mineral element contents in the leaves of ‘*Orah*’ seedlings are summarized in Table 4. Comparing organic fertilizer with only chemical fertilizers or no fertilizers at all, the levels of P and Mg remained relatively stable across all treatments, while the remaining eight elements exhibited significant increases. Among the different treatments, the organic fertilizer group exhibited the highest leaf contents of calcium (Ca) (25.73 g/kg) and boron (B) (96.67 g/kg), while seedlings treated with bio-organic fertilizer resulted in the highest levels of K (15.2 g/kg) and copper (Cu) (21.59 mg/kg) across the three concentrations tested. Additionally, ‘*Orah*’ seedlings treated with rapeseed cake showed elevated leaf contents of Fe (95.17 mg/kg), Mn (37.8 mg/kg), and Zn (16.59 mg/kg) compared to other treatments. Furthermore, variations in fertilization concentration within the same fertilizer type led to differences in mineral elements in the seedling leaves. Specifically, Fe and Zn contents significantly increased from D1 to D2 in the rapeseed cake treatment but decreased at D3, even falling below control levels. Mn content in ‘*Orah*’ seedling leaves also decreased as fertilizer concentration increased (D1 > D2 > D3). Notably, of the four organic fertilizers tested, rapeseed cake fertilizer significantly enhanced four mineral nutrients in the leaves.

### 3.2. Variations in Chemical and Biological Properties of Orchard Soils Following the Application of Different Organic Fertilizers

#### 3.2.1. Effects of Different Organic Fertilizer on Soil Enzyme Activities

The application of organic fertilizer significantly enhanced the activity of four enzymes in the soil (Table 5). Compared to only chemical fertilizers or no fertilizers at all, the soils treated with organic fertilizers showed substantially more dehydrogenase and phosphatase, followed by urease. In contrast, the increase in soil sucrase activity was relatively modest. Furthermore, different types of organic fertilizer treatments resulted in varying degrees of enhancement in soil enzyme activity. The dehydrogenase activity in the A3 treatment was the highest, while the soil phosphatase activity in the B3 treatment was the highest. The D1 treatment demonstrated the highest sucrase activity, and the D3 treatment showed significantly higher urease activity compared to the other treatments.

#### 3.2.2. Effect of Different Organic Fertilizers on Soil Mineral Elements

The analysis of soil chemical and biological properties (Table 6) indicated a substantial increase in soil organic matter (SOM) content following the application of organic fertilizer. Compared to the control group (CK1), the SOM content in the B3 treatment increased by a notable 321.21%. Compared with chemical only fertilizers or no fertilizers at all, the various types of organic fertilizers had distinct effects on the mineral element composition of the soil. For instance, the common organic fertilizer effectively increased the concentrations of P, Zn, and B in the soil, while the bio-organic fertilizer enhanced the K, Ca, and Mg contents. Rapeseed cake, on the other hand, significantly elevated N, Cu, Fe, and Mn concentrations in the soil. Specifically, the Fe and Mn contents in the rapeseed cake group were 2.75 and 2.87 times higher than those in the control group, respectively. Meanwhile, the K content in the bio-organic fertilizer group was 1.82 times higher than that of the control.

**Figure 1:**
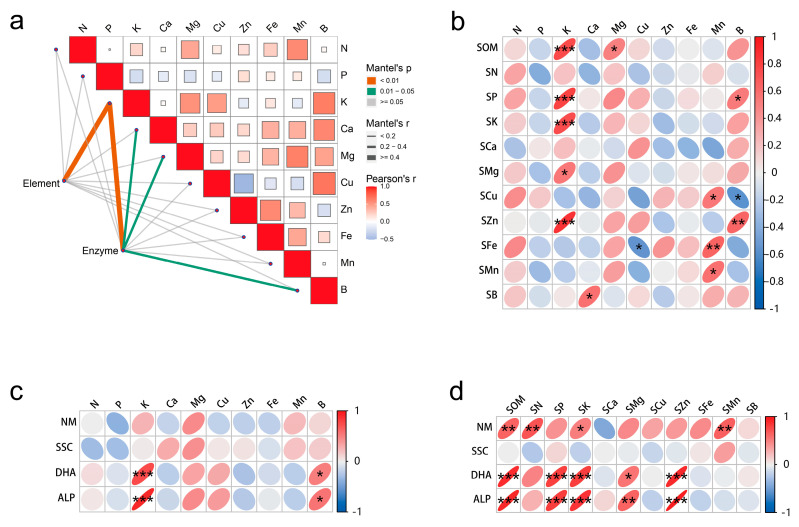
Correlation diagram of leaf elements, soil elements and soil enzyme activity. (**a**) Comprehensive correlation of soil elements, soil enzyme activity and leaf elements; (**b**) correlation of soil elements and leaf elements; (**c**) correlation of soil enzyme activities and leaf elements; (**d**) correlation of soil elements and soil enzyme activity. Element in a is soil elements, Enzyme in (**a**) is soil enzyme activity, ‘s’ before the element name indicates the element in the soil; NM, soil urease; SSC, soil sucrase; DHA, soil dehydrogenase; ALP, soil phosphatase. *, ** and *** represents the difference is significant at *p* < 0.05, *p* < 0.01 and *p* < 0.001 level.

The correlation analysis revealed a significant association between the leaf elements, soil elements, and soil enzyme activity. In particular, the K content in the leaves showed a strong correlation with soil elements and soil enzyme activity, while Ca, Mg, and B in the leaves were significantly correlated with soil enzyme activity (Figure 1a). More specifically, soil SOM, P, K, and Zn exhibited a positive correlation with K in leaves (*p* < 0.001). Fe in the soil was positively correlated with Mn in the leaves (*p* < 0.01); and Zn in the soil was positively correlated with B in the leaves (*p* < 0.01) (Figure 1b). Soil dehydrogenase (DHA) and soil phosphatase (ALP) were significantly positively associated with K in the leaves (*p* < 0.001), as well as with B in the leaves (*p* < 0.05) (Figure 1c). As illustrated in Figure 1d, the soil elements were strongly positively correlated with DHA and ALP, followed by urease; however, the relationship between sucrase and soil elements was weak. Soil SOM, P, K, and Zn were significantly positively correlated with DHA and ALP (*p* < 0.001), while Mg showed a positive correlation only at the *p* < 0.05 level. Soil SOM, N and Mn were significantly positively correlated with urease activity (*p* < 0.01).

### 3.3. Impact of Different Organic Fertilizer Applications on the Composition and Structure of Soil Bacterial Communities in Citrus Orchards

#### 3.3.1. Effect of Different Organic Fertilizer Applications on Soil Bacterial Community Composition

**Figure 2:**
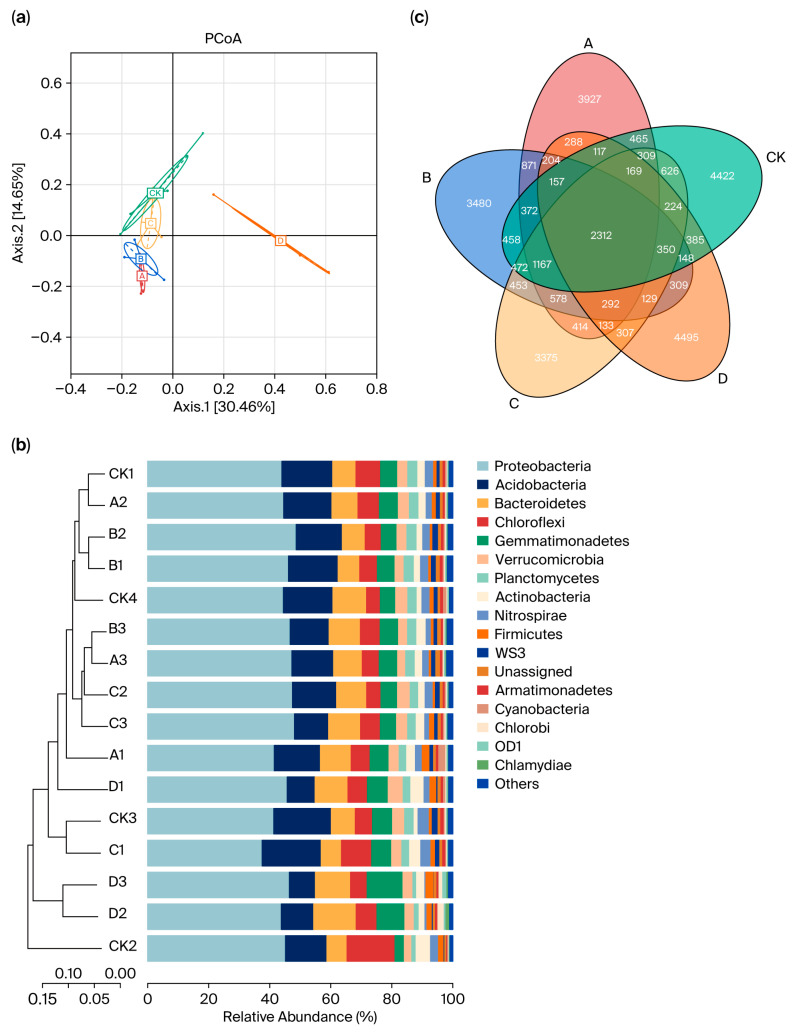
Bacterial community composition and species characteristics of different organic fertilizer treatments. (**a**) Principal coordinates analysis (PCoA) of beta diversity based on classification of the OTUs at a dissimilarity level of 0.03 for individual samples relative to the treatments. (**b**) Species abundance cluster map. (**c**) Venn diagram of species abundance in soil with different treatments. Note: A, common organic fertilizer; B, bio-organic fertilizer; C, sheep manure; D, rapeseed cake; CK, control group with or without fertilizer. The same as below.

The PCoA analysis highlighted distinct community characteristics of the soil bacteria influenced by the five different fertilizer treatments. Treatments A, B, and C showed similar bacterial community structures, whereas the CK group was relatively distinct, although it did not form clearly separated clusters. In contrast, treatment D exhibited significant divergence and formed a separate cluster (Figure 2a). At the phylum level, 17 species had a relative abundance greater than 1%, with the top five bacteria being *Proteobacteria*, *Acidobacteria*, *Bacteroides*, *Chloroflexi* and *Gemmatimonadetes*. Clustering analysis demonstrated noticeable differences in the composition of soil bacterial communities across the various treatments (Figure 2b). Notably, the common organic fertilizer and bio-organic fertilizer displayed the highest similarity in bacterial species composition. In comparison, the rapeseed cake fertilizer significantly differed from the first three fertilizers in terms of bacterial composition. Additionally, similar effects on biological composition were observed between the low and medium fertilizer concentrations. The Venn diagram illustrates that 2312 OTUs were commonly shared between the four fertilizer groups and the control group (Figure 2c), with the most unique OTUs in the rapeseed cake group and the least at 3375 in the sheep manure group.

#### 3.3.2. Effect of Different Organic Fertilizer Applications on Soil Bacterial Community Structure

**Figure 3:**
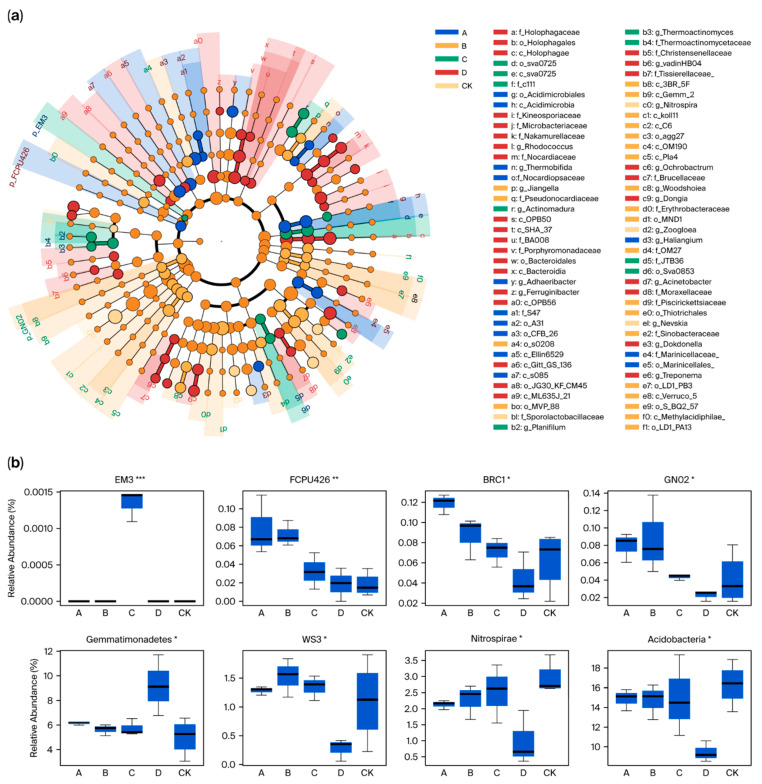
Analysis of different microbial species under different treatments. (**a**) LEfSe analysis to explore the difference in microbial community structure between treatments. (**b**) Major differentiating species in different treatments. *, ** and *** represents the difference is significant at *p* < 0.05, *p* < 0.01 and *p* < 0.001 level.

The linear discriminative analysis (LDA)-based efficiency measurement method (Lefse) is a powerful tool for high-dimensional classification and comparison, and is widely used to identify species that are most likely to contribute to differences between groups. As illustrated in Figure 3a, significant variations were observed in the species composition between the five groups, with a total of 19 classes showing notable distinctions. Among them, group A included two classes, Acidimicrobiia and S085. Group B contained eight classes, such as Methylacidiphilae. Group C contained one class Sva0725. Group D had eight classes, such as Bacteroidia, while the CK group did not exhibit any specific class. Using a *p* value < 0.05 as the threshold for statistical significance, we detected species with significant variations at the phylum level across the five groups and generated a corresponding box plot (Figure 3b). The results showed that there were significant differences in eight species among the five groups, with Acidobacteria exhibiting the highest average relative abundance at 14.08%, followed by Gemmatimonadetes at 6.35%, and EM3 showing the lowest abundance at 0.0013%. Additionally, group D displayed significantly different abundance levels compared to groups A, B, and C. For instance, the abundance of Gemmatimonadetes in group D was approximately 1.58 times higher than the average of the other three groups. Conversely, the average abundances of FCPU426, BRC1, WS3, Nitrospirae, Acidobacteria and GNO2 in groups A, B, and C were 3.30, 2.10, 5.02, 2.33, 1.58 and 3.14 times higher than those in group D, respectively.

### 3.4. Analysis of the Direct Relationships Between Soil Bacteria and Soil Chemical and Biological Indicators

#### 3.4.1. Relationships Between Bacterial Community and Soil Chemical Components

**Figure 4:**
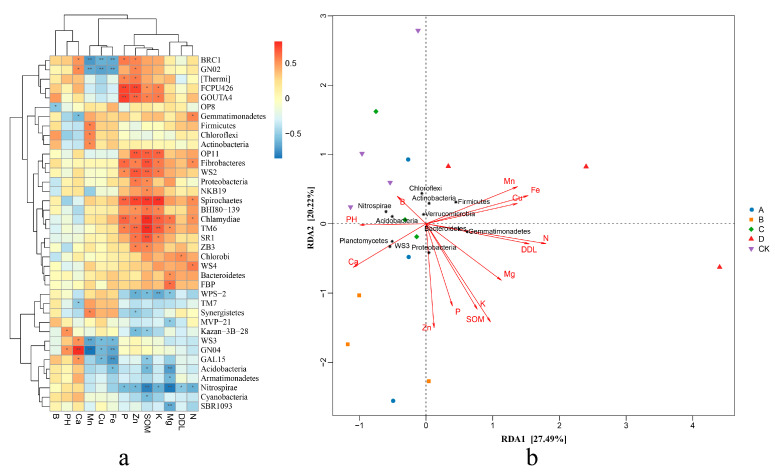
Correlation analysis between microorganisms and soil mineral nutrition under different treatments. (**a**) Cluster correlation analysis of soil chemical and biological indices and main flora. (**b**) RDA analysis of soil chemical and biological indices and main flora. * and ** represents the difference is significant at *p* < 0.05 and *p* < 0.01 level.

This study identified 16 species that were significantly associated with specific environmental factors, using the correlation coefficient |cor| > 0.5 and *p* < 0.01 as the threshold. In group A, *FCPU426* showed a strong positive correlation with P and Zn, while *GOUTA4* was significantly positively correlated with P, *OP11* with Zn, SOM and K; and *TM6* demonstrated a significant positive association with SOM. In group B, *GNO4* displayed a significant positive correlation with Ca, and *WS2* was significantly positively correlated with Zn and SOM. In group C, WPS-2 exhibited a notable positive correlation with K. In contrast, *BRC1* and *GNO2* were significantly negatively correlated with Mn, Cu, and Fe in group D, while *WS3* showed a significant negative correlation with Mn. Additionally, *GNO4* was significantly negatively correlated with Mn and Fe, *Acidobacteria* exhibited a significant negative correlation with Mg; *Nitrospirae* was significantly negatively correlated with SOM and Mg; *SBR1093* was significantly negatively correlated with Mg; *OP11* showed a significant positive correlation with Zn, SOM and K; *Fibrobacteres* exhibited a significant positive correlation with SOM; and *Spirochaetes* showed a significant positive correlation with Zn, SOM and K. *Chlamydiae* demonstrated a significant positive correlation with P, SOM and K, while *TM6* was significantly positively correlated with SOM (see Figure 4).

Redundancy analysis of the bacterial species and the soil mineral elements revealed that the influence of environmental factors on species distribution followed this order: N > K > Fe > Zn > conductivity (DDL) > SOM > Mn > Mg > Cu > P > Ca > pH > B. Among the eleven bacterial species with high relative abundance and a strong environmental correlation, soil mineral elements significantly influenced nine of them. Specifically, Acidobacteria showed a significant negative correlation with SOM, Mg, and Fe; Actinobacteria, Chloroflexi, and Firmicutes exhibited a significant positive correlation with Mn; Bacteroidetes was significantly positively correlated with Mg; Gemmatimonadetes demonstrated a significant positive correlation with N but a negative correlation with Ca; Nitrospirae had a significant negative correlation with conductivity, SOM, N, P, K, and Mg; Proteobacteria was significantly positively correlated with SOM and Zn; and WS3 was significantly negatively correlated with Ca and significantly negatively correlated with Cu, Fe, and Mn. Based on the correlation between the samples and the bacterial species, the overlapping similarity of species compositions in groups A, B, C, and CK were very similar, whereas group D was more distant from the other groups. Group D was significantly positively correlated with Bacteroidetes, Gemmatimonadetes, and Firmicutes, and significantly negatively correlated with Acidobacteria, Nitrospirae, and WS3. In contrast, the relative abundance of Proteobacteria, Actinobacteria, and Chloroflexi in group D did not show significant differences compared to the other groups.

#### 3.4.2. Relationships Between Bacterial Community and Soil Enzymes

**Figure 5:**
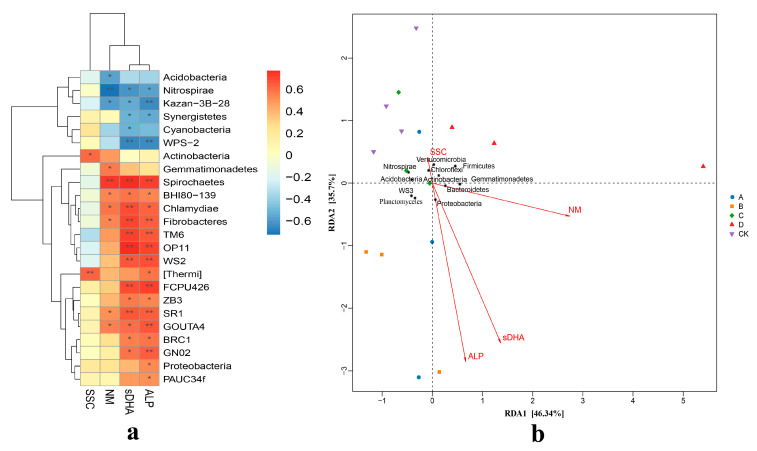
Correlation analysis of microorganisms and soil main enzymes under different treatments. (**a**) Cluster correlation analysis of soil enzyme activity and main flora. (**b**) RDA analysis of soil enzyme activity and main flora. * and ** represents the difference is significant at *p* < 0.05 and *p* < 0.01 level.

The heatmap revealed that a total of 24 species were highly correlated with soil enzyme activities, with 14 species showing significant correlations. In group A, *FCPU426*, *OP11*, and *TM6* exhibited significant positive correlations with both soil dehydrogenase and acid phosphatase, *GOUTA4* showed a significant positive correlation specifically with acid phosphatase, while [*Thermi*] was significantly positively correlated with soil sucrase. In group B, *WS2* and *SR1* were significantly positively correlated with both soil dehydrogenase and acid phosphatase, *GN02* was significantly positively correlated with acid phosphatase, whereas *Kazan-3B-28* and *WPS-2* were significantly negatively correlated with dehydrogenase and acid phosphatase, respectively. In group D, *Nitrospirae* showed a significant negative correlation with urease; *Spirochaetes* was significantly positively correlated with soil urease, dehydrogenase, and acid phosphatase; *Chlamydiae* was significantly positively correlated with dehydrogenase; and *Fibrobacteres*, *OP11*, and *TM6* also displayed significant positive correlations with dehydrogenase and acid phosphatase (see Figure 5).

### 3.5. Functional Prediction of Bacteria Communities Following the Application of Different Types of Organic Fertilizer

**Figure 6:**
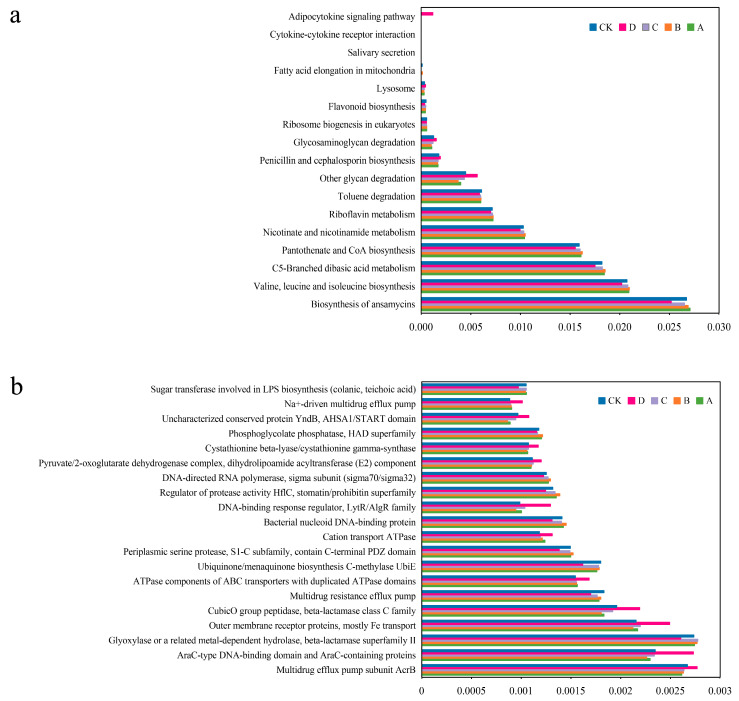
Prediction of soil bacterial function under different amendment treatments. (**a**) Clusters of orthologous groups of metabolic pathway (KEGG) of different treatments. (**b**) Clusters of orthologous groups of proteins (COG) of different treatments.

Based on the 16S rDNA sequencing data, PICRUSt was employed to predict the metabolic function of bacterial communities under the various fertilization treatments. A total of 193 sub-functions were annotated at the KEGG level, of which 17 exhibited significant differences in all the groups (*p* < 0.05), which were divided into three categories: metabolism, genetic information processing, and environmental information processing (Figure 6a). Metabolism constituted the predominant function in each treatment group; however, notable variations were observed in the relative abundance of specific functional categories among the groups. In group D, fewer functional genes were associated with ansamycin biosynthesis, valine, leucine, and isoleucine biosynthesis; C5-branched dicarboxylic acid metabolism, pantothenate and CoA biosynthesis, nicotinic acid and nicotinamide metabolism, riboflavin metabolism and toluene degradation compared to the other fertilization treatments. Conversely, more functional genes related to other glycan degradation, penicillin and cephalosporin biosynthesis, glycosaminoglycan degradation, and adipocytokine signaling pathway were more abundant in group D than in the other groups.

The COG functional annotation analysis found that the expression levels of 18 proteins (enzymes) in the fertilization groups were significantly higher than those in the control group (Figure 6b). Among them, group A was more involved in the biosynthesis of lipopolysaccharide glycosyltransferase. In group B, the proteins with high expression included serine protease, the bacterial DNA binding protein, active factor HflC regulatory protein, directional RNA polymerase, phosphoglycolic acid phosphatase and others. The expression level of acetaldehyde or related metal-dependent hydrolase in group C was higher than those in the other groups. In addition, significantly elevated expression levels of 11 proteins were observed in group D, which were primarily associated with ATP synthesis, drug resistance, glycolipid synthesis, Fe transport, and so on.

## 4. Discussion

### 4.1. Effects of Different Organic Fertilizers on Growth and Leaf Elements of Citrus

Previous studies have demonstrated that the utilization of an organic fertilizer can enhance both the soil’s physical structure and fertility more than the sole application of chemical fertilizer [30], thereby improving the water and fertilizer utilization efficiency, increasing crop yield, and improving crop quality [31]. This is consistent with the findings of our study. The increase in stem diameter, plant height, and number of terminal branches of citrus seedlings was promoted to varying degrees by four types of organic fertilizer. This is attributed to the presence of essential macro and trace mineral elements as well as rich organic nutrients in the organic fertilizers. Additionally, they improve the soil’s physical and chemical properties, thereby enhancing soil fertility [32,33,34] and providing a good soil environment and nutritional conditions for tree growth. It was observed that each organic fertilizer treatment increased the content of elements in the citrus leaves, with rapeseed cake being the most obvious, especially the contents of Fe, Mn, and Zn. N is a component of chlorophyll, and Fe, Mn, and Zn have catalytic functions in chlorophyll synthesis. This experiment supposed that rapeseed cake fertilizer can help the chlorophyll synthesis of citrus seedlings, enhance the photosynthetic efficiency of plants, and promote plant growth.

At a low fertilization concentration (10 g/kg) of the four organic fertilizers, the growth parameters exhibited a significant increase compared to those at the other two concentrations. This finding is consistent with previous studies. Gao et al. demonstrated that organic fertilizer could achieve optimal effects in improving soil and promoting seedling growth only at a suitable fertilization concentration, which was lower than the recommended application rate [35]. Further increasing the amount of organic fertilizer may result in an inhibitory effect on seedling growth. Moreover, there are discernible variations in the nutrient characteristics of the different organic fertilizers. The mineralization process of organic fertilizers is influenced by multiple factors such as temperature, water availability, microbial species, and the inherent physicochemical properties. Consequently, the performance of different organic fertilizers varies under different potting conditions [36].

### 4.2. Effects of Different Organic Fertilizers on Soil Physical and Chemical Properties

The physical and chemical properties of soil are important factors influencing crop growth. Favorable soil conditions facilitate the enhancement and release of soil-available nutrients [37]. Utilizing organic fertilizer not only adjusts the physical and chemical properties of the soil but also enhances soil nutrition, particularly SOM, which has been widely recognized by the industry [38]. The findings of this study revealed that applying 100 g/kg bio-organic fertilizer led to a remarkable increase of 321.21% in the SOM content. The common organic fertilizer effectively enhances the contents of P, Zn, and B in the soil, while the bio-organic fertilizer can increased the contents of K, Ca and Mg. Moreover, rapeseed cake significantly raised the N, Fe, and Mn contents in the soil by 2.75 times for Fe and 2.87 times for Mn compared to the control group. We speculate that this is due to the difference in nutrient characteristics of the different organic fertilizers. Rapeseed cake demonstrates superior advantages over the other organic fertilizers with significant increases in N as well as Fe and Mn. These elements play a vital role in DNA synthesis, respiration, and photosynthesis [39]. Furthermore, the application of organic fertilizer can enhance soil enzyme activity, and this is widely acknowledged [40]. Our research indicates that treatment with organic fertilizer leads to increased activities of dehydrogenase, phosphatase, sucrase, and urease, which is consistent with previous studies [41,42]. The activity levels of sucrase and urease in the rapeseed cake treatment were significantly higher than those in other treatments. These two enzymes are known to promote plant sugar and nitrogen absorption [43]. We anticipate that the application of rapeseed cake may not only promote vegetative growth, but also improve the quality of citrus.

### 4.3. Effects of Different Organic Fertilizers on Soil Bacterial Composition and Abundance

The diversity and composition of soil microbial communities play a pivotal role in maintaining soil function and ecological stability [44,45]. Numerous studies have demonstrated that bacterial diversity in crop soils are more bacterially diverse following the application of organic fertilizers compared to chemical fertilizers [46,47,48,49]. Sharaf et al. [43] also reported that organic fertilizer application alters the community diversity and structure of soil bacteria and fungi in apple orchards, leading to increased bacterial diversity and an improved soil microbial environment [50]. In this study, high-throughput sequencing technology was employed to examine the soil microbial community subsequent to treatment with four different types of organic fertilizers. The results revealed substantial variations in the relative abundance of dominant bacterial taxa across the different fertilizer treatments. The cluster analysis indicated that the soil bacterial composition in the rapeseed cake group was markedly distinct from those in the other three fertilizer groups. The amount of *Gemmatimonadetes* in group D was 1.58 times higher than the average of groups A, B and C, while the mean values of *FCPU426*, *BRC1*, *WS3*, *Nitrospirae*, *Acidobacteria*, and *GNO2* in groups A, B, C were 3.30, 2.10, 5.02, 2.33, 1.58 and 3.14 times greater than those of group D, respectively. Previous studies on tea and peach planting indicate that the application of rapeseed cake could increase the abundance of beneficial bacteria in soil [51,52,53].

### 4.4. The Function of Different Microorganisms in Different Organic Fertilizer Treatments

Compared with plant growth biomass, the soil bacterial community structure was more significantly influenced by the different fertilizer treatments [54,55]. The richness of the microbial community serves as a key indicator of soil microbial ecological functions and can regulate and indicate changes in soil microbial functional genes [56]. Discrepancies in bacterial populations result in variations in bacterial function. Previous studies have shown that utilizing the PICRUSt tool for functional prediction analysis aids in assessing microbial metabolic characteristics [57]. In this experiment, the same approach was applied to predict the bacterial functions within each organic fertilizer group, revealing that these functions could be categorized as metabolism, genetic information processing and environmental information processing. Although the relative abundances of different functional genes varied across the four fertilization groups, metabolic function remained the most important core function of the bacterial community. Bacteria participate in material circulation and transformation within soil through their metabolic activities. Xia et al. [48] found that the application of biochar and potassium fertilizer positively altered the functional genes in soil bacteria related to ‘amino acid transport and metabolism’ and ‘energy production and transformation’ through functional prediction analysis. Similarly, biochar was shown to increase glucose metabolism in bacteria while decreasing the abundance of saprophytic fungi [58]. The quantities of *Gemmatimonadetes*, *FCPU426*, *BRC1*, *WS3*, *Nitrospirae*, *Acidobacteria*, and *GN02* were significantly different between the treatments in this study. *Gemmatimonadetes*, *Nitrospirae* and *Acidobacteria* were widely distributed in the natural microbial community and were one of the nine most abundant phyla in the soil [59,60,61]. They are not only adaptable to drought but also to oligotrophic conditions [62]. Some strains have been reported for their ability to perform photosynthesis and reduce the amount of N_2_O emitted [63]. The correlation analysis in this study showed that *Gemmatimonadetes* was significantly positively correlated with N. The *Nitrospirae* genus consists of typical nitrite-oxidizing bacteria (NOB) and ammonia-oxidizing microorganisms, and plays a crucial role in nitrogen cycling. Ammonia-oxidizing bacteria convert ammonia into nitrite which is subsequently converted into nitrate by nitrite-oxidizing bacteria, making it available for plant uptake [61]. Acidobacteria represents an under-represented phylum of soil bacteria with members widely distributed in almost all ecosystems. Several studies have indicated that *Acidobacteria* is plant growth-promoting rhizobia [64]. Kielak et al. [65] have found that three strains of *Granulicella* and *Acidicapsa* are active producers of the plant hormone IAA and siderophores. All three strains can adhere to the root system, form a biofilm, and colonize along the root surface. This study found that the relative abundance of *Acidobacteria* in the rapeseed cake treatment was lower than that in other treatments, and it was negatively correlated with Fe, which may be the result of the response of *Acidobacteria* to the high-iron-content environment of the rapeseed cake group.

## 5. Conclusions

The application of an appropriate concentration of common organic fertilizer, bio-organic fertilizer, sheep manure and rapeseed cake could significantly increase the growth of citrus seedlings, increase soil mineral element content and soil enzyme activity level. However, the effects and degrees of influence vary among the four kinds of organic fertilizers. The soil-specific microorganisms of the citrus seedlings treated with rapeseed cake fertilizer were more numerous, and the proportion of the top 10 species in terms of abundance also demonstrates significant differences between the rapeseed cake fertilizer and the other three types of organic fertilizers. The correlation analysis between the composition of the microbial community and the soil chemical and biological indexes showed that the core microbial community in the rapeseed cake fertilizer promoted the synthesis and transport of sugar, Fe and other substances, and thus increased the growth of ‘*Orah*’. Based on these findings, we suggest that the application of rapeseed cake fertilizer is preferred to promote citrus growth and increase the quantities of nutrients and microorganisms in citrus orchards.

## Figures and Tables

**Table 1:** Experimental design.

Category	Number	Fertilizer Treatment	Application Rate (g/kg)	Spraying with 5 ‰ Urea (times/month)
Application of organic fertilizer	1	Organic–inorganic fertilizer	10	2
	2		50	2
	3		100	2
	4(A1)	Common organic fertilizer	10	2
	5(A2)		50	2
	6(A3)		100	2
	7(B1)	Bio-organic fertilizer	10	2
	8(B2)		50	2
	9(B3)		100	2
	10(C1)	Sheep manure	10	2
	11(C2)		50	2
	12(C3)		100	2
	13(D1)	Rapeseed cake	10	2
	14(D2)		50	2
	15(D3)		100	2
No application of organic fertilizer	16(CK1)	Compound fertilizer	25 g/plant/month	2
	17(CK2)	Urea	25 g/plant/month	2
	18(CK3)	-	0	2
	19(CK4)	-	0	0

**Table 2:** Nutrient conditions of various fertilizers and citrus orchard soil.

Types	SOM (%)	TN (g/kg)	AP (g/kg)	AK (g/kg)	Ca (g/kg)	Mg (g/kg)	Cu (mg/kg)	Zn (mg/kg)	Fe (mg/kg)	Mn (mg/kg)	B (mg/kg)
Organic–inorganic fertilizer	14.41 ± 0.04	9.67 ± 0.07	0.26 ± 0	1.5 ± 0.01	0.23 ± 0	3.56 ± 0	1.52 ± 0.01	157.86 ± 0.57	147.25 ± 0.14	146.55 ± 3.01	26.1 ± 0.88
Common organic fertilizer	53.21 ± 0.24	8.02 ± 0.07	0.22 ± 0	1.56 ± 0	1.27 ± 0	4.58 ± 0.01	1.15 ± 0.01	34.75 ± 0.51	43.81 ± 1.05	41.99 ± 1.32	17.28 ± 0.59
Bio-organic fertilizer	52.42 ± 0.11	8.84 ± 0.08	0.22 ± 0.01	1.52 ± 0	1.15 ± 0.02	4.65 ± 0.01	0.66 ± 0.01	29.79 ± 0.34	41.73 ± 0.7	38.58 ± 0.88	30.78 ± 0.9
Sheep manure	82.55 ± 0.43	8.48 ± 0.07	0.17 ± 0	1.49 ± 0	0.44 ± 0.01	4.61 ± 0.01	0.21 ± 0.01	8.51 ± 0.18	2.37 ± 0.86	21.19 ± 3.6	26.36 ± 0.31
Rapeseed cake	27.46 ± 0.27	17.12 ± 0.12	0.13 ± 0	1.34 ± 0.01	1.52 ± 0.01	4.62 ± 0.01	0.65 ± 0.03	13.63 ± 0.11	162.26 ± 0.48	49.16 ± 0.15	3.23 ± 0.13
Compound fertilizers	0 ± 0	8.24 ± 0.07	0.62 ± 0.01	1.21 ± 0.01	0.08 ± 0	0.02 ± 0	0.01 ± 0	0 ± 0	0 ± 0	0 ± 0	0 ± 0
Urea	0 ± 0	452.36 ± 15.42	0 ± 0	0 ± 0	0 ± 0	0 ± 0	0 ± 0	0 ± 0	0 ± 0	0 ± 0	0 ± 0
Citrus orchard soil	1.42 ± 0.09	3.35 ± 0.48	0.04 ± 0	0.3 ± 0	2.04 ± 0.01	3 ± 0.01	0.69 ± 0.04	0.92 ± 0.04	40.3 ± 0.15	8.11 ± 0.25	37.37 ± 0.91

**Table 3:** Effects of different kinds and concentrations of organic fertilizers on the growth of citrus seedlings.

Treatment	Stem Diameter (mm)	Height (cm)	Number of Terminal Branches
A1	11.15 ± 1.23 a**	78.40 ± 10.13 a**	39.33 ± 10.72 a**
A2	8.02 ± 0.99 de**	66.12 ± 10.33 bcd**	19.44 ± 9.68 fg**
A3	9.20 ± 0.74 c**	66.40 ± 4.83 bcd**	28.89 ± 10.17 bcdef**
B1	10.36 ± 1.1 ab**	74.02 ± 6.63 ab**	37.78 ± 5.04 ab**
B2	9.24 ± 1.42 c**	68.61 ± 5.19 bc**	25.44 ± 14.93 cdefg**
B3	9.19 ± 0.86 c**	62.80 ± 7.79 cd**	33 ± 10.33 abcd**
C1	10.53 ± 1.27 ab**	70.09 ± 6.91 bc**	35.56 ± 9.10 abc**
C2	9.16 ± 1.04 c**	67.13 ± 4.04 bc**	26.67 ± 11.57 cdefg**
C3	9.64 ± 0.7 bc**	70.71 ± 6.14 bc**	30.22 ± 5.38 abcde**
D1	10.71 ± 1.18 a**	69.27 ± 9.19 bc**	39.44 ± 9.80 a**
D2	8.60 ± 1.16 cde**	64.91 ± 11.97 cd**	21.22 ± 9.13 efg**
D3	7.80 ± 1.01 e**	57.97 ± 11.18 d**	17.56 ± 9.71 g**
CK1	9.33 ± 0.86 c**	67.29 ± 5.25 bc**	26.44 ± 13.52 cdefg**
CK2	8.62 ± 0.88 cde**	65.63 ± 8.90 bcd**	29.11 ± 6.35 bcdef**
CK3	8.90 ± 1.08 de**	67.33 ± 5.84 bc**	24.22 ± 7.05 defg**
CK4	9.16 ± 0.78 c**	63.71 ± 8.93 cd**	22.00 ± 3.90 efg**

A, B, C, and D represent common organic fertilizer, bio-organic fertilizer, sheep manure and rapeseed cake fertilizer, respectively. Numbers 1, 2, and 3 after A, B, C, and D for application concentrations of 10 g/kg (1%), 50 g/kg (5%) and 100 g/kg (10%), respectively. CK1: monthly applications of 25 g compound fertilizer, CK2: monthly applications of 25 g urea, CK3: only 5 ‰ urea twice per month, CK4: no fertilizers. Lowercase letters after numbers in the same column represent the difference was significant at *p* < 0.05 level. ** represent the difference is significant at *p* < 0.01 levels.

**Table 4:** Effects of different organic fertilizers treatments on mineral elements in citrus leaves.

Treatment	N g/kg	P g/kg	K g/kg	Ca g/kg	Mg g/kg
A1	23.55 ± 2.03	1.28 ± 0.036 f**	13.76 ± 1.02 abc**	23.65 ± 2.34 ab**	2.10 ± 0.22 ab*
A2	22.69 ± 1.98	1.41 ± 0.052 cde**	13.58 ± 0.99 abcd**	25.73 ± 2.01 a**	2.13 ± 0.23 ab*
A3	24.38 ± 2.53	1.54 ± 0.048 ab**	14.28 ± 1.21 ab**	17.52 ± 1.98 efg**	2.11 ± 0.19 ab*
B1	24.13 ± 2.14	1.41 ± 0.037 cde**	13.14 ± 0.89 bcde**	19.79 ± 2.03 cdef**	2.12 ± 0.24 ab*
B2	25.52 ± 3.01	1.40 ± 0.049 cde**	13.60 ± 0.96 abcd**	19.124 ± 1.87 cdef**	2.24 ± 0.19 ab*
B3	22.93 ± 2.36	1.46 ± 0.068 bcd**	15.20 ± 0.87 a**	18.48 ± 1.96 def**	2.17 ± 0.2 ab*
C1	22.10 ± 2.14	1.32 ± 0.051 ef**	12.37 ± 0.98 cdef**	20.60 ± 2.05 bcdef**	1.94 ± 0.24 bc*
C2	23.40 ± 1.98	1.42 ± 0.055 cd**	11.81 ± 0.88 def**	20.34 ± 1.88 bcdef**	1.98 ± 0.19 abc*
C3	25.56 ± 2.58	1.43 ± 0.047 cd**	13.20 ± 0.73 bcde**	21.59 ± 1.64 bcd**	2.40 ± 0.21 a*
D1	22.81 ± 2.35	1.41 ± 0.061 cde**	12.25 ± 0.81 cdef**	22.64 ± 1.92 abc**	2.40 ± 0.27 a*
D2	26.36 ± 3.33	1.40 ± 0.039 cde**	12.75 ± 0.92 bcdef**	20.10 ± 2.41 bcdef**	2.17 ± 0.23 ab*
D3	24.07 ± 2.71	1.36 ± 0.047 def**	12.26 ± 0.84 cdef**	14.63 ± 2.06 g**	2.09 ± 0.22 ab*
CK1	26.56 ± 1.69	1.49 ± 0.061 abc**	11.65 ± 1.05 ef**	22.36 ± 1.87 abc**	1.90 ± 0.25 bc*
CK2	22.20 ± 3.04	1.58 ± 0.075 a**	11.02 ± 0.96 def**	21.17 ± 1.88 bcde**	1.93 ± 0.2 abc*
CK3	23.33 ± 1.88	1.56 ± 0.059 a**	11.81 ± 0.87 f**	19.81 ± 1.67 cdef**	2.02 ± 0.19 bc*
CK4	20.93 ± 2.01	1.45 ± 0.049 bcd**	11.27 ± 0.91 f**	17.14 ± 2.05 fg**	1.65 ± 0.18 c*
Treatment	Cu mg/kg	Fe mg/kg	Mn mg/kg	Zn mg/kg	B mg/kg
A1	13.64 ± 2.34 c**	60.53 ± 7.68 fgh**	19.79 ± 2.11 e**	13.29 ± 1.26 cde**	72.03 ± 6.78 b**
A2	14.69 ± 2.01 bc**	80.71 ± 7.94 bcd**	26.10 ± 2.35 c**	13.26 ± 1.32 cde**	96.67 ± 8.36 a**
A3	13.27 ± 1.98 c**	68.29 ± 8.02 def**	20.00 ± 2.03 e**	14.30 ± 1.2 bcd**	61.06 ± 6.12 c**
B1	21.59 ± 2.03 a**	52.01 ± 6.48 gh**	23.07 ± 2.16 cde**	11.71 ± 1.26 e**	63.49 ± 6.01 bc**
B2	15.39 ± 1.87 b**	75.90 ± 5.39 bcde**	24.76 ± 2.58 cd**	11.46 ± 1.06 e**	73.56 ± 6.89 b**
B3	8.25 ± 1.96 d**	71.03 ± 7.06 cdef**	21.20 ± 2.36 de**	13.76 ± 1.07 cde**	63.30 ± 5.87 bc**
C1	7.71 ± 2.05 d**	85.88 ± 8.02 ab**	19.37 ± 1.96 e**	14.66 ± 1.22 bcd**	49.28 ± 5.02 de**
C2	7.63 ± 1.88 d**	61.79 ± 7.77 fgh**	21.42 ± 1.97 de**	16.35 ± 1.29 ab**	53.97 ± 5.22 cd**
C3	6.61 ± 1.64 def**	83.43 ± 9.12 abc**	35.32 ± 2.06 ab**	17.61 ± 1.35 a**	61.25 ± 6.23 c**
D1	7.00 ± 1.92 de**	71.48 ± 8.23 cdef**	37.80 ± 3.14 a**	13.92 ± 1.45 cde**	57.81 ± 5.78 cd**
D2	5.80 ± 2.41 efg**	95.17 ± 4.56 a**	33.26 ± 3.02 b**	16.59 ± 1.62 ab**	49.61 ± 4.85 de**
D3	4.39 ± 2.06 g**	49.78 ± 6.36 h**	25.12 ± 2.95 cd**	12.18 ± 1.38 de**	42.55 ± 4.71 e**
CK1	7.35 ± 1.87 de**	64.60 ± 5.14 efg**	33.82 ± 2.55 ab**	13.35 ± 1.25 cde**	47.19 ± 5.23 de**
CK2	7.89 ± 1.88 de**	73.61 ± 6.14 abc**	26.52 ± 2.47 cd**	14.41 ± 1.36 bc**	49.62 ± 4.19 de**
CK3	6.85 ± 1.67 d**	83.15 ± 6.68 bcdef**	25.19 ± 2.31 c**	14.94 ± 1.41 bcd**	47.33 ± 4.36 de**
CK4	5.15 ± 2.05 fg**	51.00 ± 7.03 h**	14.01 ± 2.06 f**	13.06 ± 1.06 cde**	41.63 ± 4.01 e**

A, B, C, and D represent common organic fertilizer, bio-organic fertilizer, sheep manure and rapeseed cake fertilizer, respectively. Numbers 1, 2, and 3 after A, B, C, and D for application concentrations of 10 g/kg (1%), 50 g/kg (5%) and 100 g/kg (10%), respectively. CK1: monthly applications of 25 g compound fertilizer, CK2: monthly applications of 25 g urea, CK3: only 5 ‰ urea twice per month, CK4: no fertilizers. Lowercase letters after numbers in the same column represent the difference was significant at *p* < 0.05 level. * and ** represent the difference is significant at *p* < 0.05 level and *p* < 0.01 levels, respectively.

**Table 5:** Effects of organic fertilizers on soil enzyme activities.

Treatment	Urease μg/d/g	Sucrase mg/d/g	Dehydrogenase μg/d/g	Phosphatase nmol/h/g
A1	568.54 ± 56.14 cd**	19.98 ± 1.84 b**	531.63 ± 49.23 c**	604.34 ± 55.33 de**
A2	647.46 ± 67.21 c**	16.46 ± 1.59 cd**	835.51 ± 65.62 b**	831.43 ± 67.36 c**
A3	583.42 ± 60.03 cd**	16.97 ± 1.46 bcd**	973.92 ± 87.36 a**	1015.5 ± 84.12 b**
B1	323.97 ± 39.25 fgh**	17.22 ± 1.62 bcd**	449.73 ± 39.11 de**	553.68 ± 48.74 ef**
B2	383.99 ± 31.06 efg**	17.04 ± 1.58 bcd**	502.86 ± 47.36 cd**	681.39 ± 71.23 d**
B3	570.66 ± 58.57 cd**	17.12 ± 1.62 bcd**	821.64 ± 62.15 b**	1284.0 ± 96.27 a**
C1	437.16 ± 42.36 e**	17.34 ± 1.87 bcd**	284.93 ± 26.88 ghi**	306.3 ± 29.14 h**
C2	349.02 ± 40.35 efgh**	19.35 ± 1.66 bc**	347.54 ± 31.25 fgh**	415.88 ± 35.78 g**
C3	534.75 ± 48.67 d**	17.06 ± 1.55 bcd**	393.9 ± 34.78 ef**	450.56 ± 41.03 g**
D1	832.48 ± 68.36 b**	23.79 ± 1.87 a**	361.24 ± 30.22 fg**	477.98 ± 44.28 ef**
D2	409.04 ± 42.31 ef**	16.02 ± 1.48 d**	423.85 ± 34.78 ef**	412.37 ± 39.28 g**
D3	1125.7 ± 89.66 a**	16.35 ± 1.6 cd**	789.49 ± 52.36 b**	661.68 ± 57.92 d**
CK1	360.59 ± 32.12 efgh**	14.78 ± 1.35 d**	267.34 ± 22.47 hi**	161.93 ± 15.23 i**
CK2	321.84 ± 29.56 fgh**	17.5 ± 1.68 bcd**	245.17 ± 28.56 i**	195.48 ± 17.89 i**
CK3	309.32 ± 25.31 gh**	16.34 ± 1.58 cd**	271.23 ± 25.99 hi**	282.28 ± 20.54 h**
CK4	269.15 ± 27.64 h**	16.13 ± 1.63 d**	246.36 ± 23.14 i**	166.47 ± 15.45 i**

A, B, C, and D represent common organic fertilizer, bio-organic fertilizer, sheep manure and rapeseed cake fertilizer, respectively. Numbers 1, 2, and 3 after A, B, C, and D for application concentrations of 10 g/kg (1%), 50 g/kg (5%) and 100 g/kg (10%), respectively. CK1: monthly applications of 25 g compound fertilizer, CK2: monthly applications of 25 g urea, CK3: only 5 ‰ urea twice per month, CK4: no fertilizers. Lowercase letters after numbers in the same column represent the difference was significant at *p* < 0.05 level. ** represent the difference is significant at *p* < 0.01 levels.

**Table 6:** Effect of organic fertilizers on soil mineral elements.

Sample	SOM%	N mg/kg	P mg/kg	K mg/kg	Ca g/kg	Mg g/kg
A1	2.21 ± 0.02 h**	84.38 ± 7.89 c**	64.74 ± 6.71 abcde*	386.65 ± 49.23 de**	2.11 ± 0.21 abc*	3.5 ± 0.32 abc*
A2	3.12 ± 0.03 d**	81.18 ± 8.01 cd**	63.89 ± 6.23 abcde*	448.65 ± 46.91 c**	1.98 ± 0.2 abc*	3.67 ± 0.35 abc*
A3	3.26 ± 0.03 c**	75.77 ± 7.91 cde**	81.27 ± 7.82 abc*	417.6 ± 38.27 cd**	1.92 ± 0.24 bc*	3.27 ± 0.31 bc*
B1	1.93 ± 0.02 j**	53.73 ± 6.02 gh**	35.98 ± 4.01 cde*	272.35 ± 29.12 f**	2.18 ± 0.19 ab*	3.3 ± 0.29 bc*
B2	2.64 ± 0.03 e**	74.49 ± 6.25 cde**	63.42 ± 6.83 abcde*	374.95 ± 35.84 de**	2.17 ± 0.21 ab*	3.78 ± 0.35 abc*
B3	4.17 ± 0.03 a**	78.98 ± 7.31 cd**	73.15 ± 7.24 abcd*	687.45 ± 59.82 a**	2.1 ± 0.23 abc*	4.1 ± 0.33 a*
C1	1.52 ± 0.03 l**	69.63 ± 6.37 def**	32.5 ± 3.47 bcde*	229.15 ± 30.33 f**	1.79 ± 0.18 c*	3.15 ± 0.34 c*
C2	2.06 ± 0.03 i**	48.27 ± 5.23 h**	44.44 ± 4.29 bcde*	234.65 ± 27.64 f**	1.97 ± 0.16 abc*	3.47 ± 0.32 bc*
C3	2.49 ± 0.02 f**	69.62 ± 7.12 def**	38.78 ± 4.81 abcde*	259.65 ± 32.1 f**	2.08 ± 0 abc*	3.53 ± 0.29 abc*
D1	2.09 ± 0.03 h**	66.26 ± 6.59 ef**	53.94 ± 4.99 abcde*	246.6 ± 28.71 f**	1.89 ± 0.21 bc*	3.44 ± 0.31 bc*
D2	2.39 ± 0.03 g**	113.23 ± 6.06 b**	60.83 ± 4.67 ab*	346.3 ± 25.47 e**	1.77 ± 0.18 c*	3.77 ± 0.36 abc*
D3	3.71 ± 0.03 b**	148.72 ± 7.12 a**	51.51 ± 4.65 a*	513.65 ± 23.64 b**	1.77 ± 0.15 c*	3.87 ± 0.33 ab*
CK1	0.99 ± 0.03 n**	73.82 ± 7.36 cde**	46.68 ± 3.92 abcde*	333.2 ± 22.99 e**	1.85 ± 0.2 bc*	3.2 ± 0.34 c*
CK2	1.48 ± 0.02 k**	52.03 ± 5.88 gh**	27.74 ± 3.87 e*	211.3 ± 26.49 f**	1.94 ± 0.19 abc*	3.33 ± 0.28 bc*
CK3	1.54 ± 0.03 jk**	59.04 ± 5.91 fgh**	30.21 ± 3.44 de*	217.6 ± 23.57 f**	2 ± 0.18 abc*	3.2 ± 0.27 c*
CK4	1.24 ± 0.02 m**	61.39 ± 4.82 fg**	27.11 ± 2.98 de*	215 ± 24.71 f**	2.29 ± 0.17 a*	3.33 ± 0.31 bc*
Sample	Cu mg/kg	Fe mg/kg	Mn mg/kg	Zn mg/kg	B mg/kg	
A1	0.8 ± 0.07 g**	26.68 ± 2.3 g**	20.24 ± 1.94 fg**	3.32 ± 0.34 d**	21.97 ± 2.02 bc**	
A2	0.85 ± 0.07 fg**	34.6 ± 2.89 fg**	22.63 ± 2.03 efg**	5.64 ± 0.29 b**	24.4 ± 1.97 b**	
A3	1.18 ± 0.09 b**	39.47 ± 3.52 ef**	18.28 ± 1.87 fg**	5.39 ± 0.31 b**	19.35 ± 2.12 cdef**	
B1	0.99 ± 0.09 cdef**	39.73 ± 3.74 ef**	23.56 ± 1.63 ef**	2.72 ± 0.51 e**	17.29 ± 2.03 efg**	
B2	0.87 ± 0.07 efg**	39.9 ± 3.69 ef**	20.26 ± 2.05 fg**	3.85 ± 0.30 c**	17.07 ± 1.84 efg**	
B3	0.92 ± 0.08 defg**	44.45 ± 4.03 e**	21.93 ± 1.88 fg**	6.95 ± 0.42 a**	17.85 ± 2.03 defg**	
C1	1.02 ± 0.07 cde**	60.33 ± 5.21 d**	36.76 ± 2.04 c**	1.91 ± 0.19 ghi**	20.56 ± 1.79 bcde**	
C2	0.94 ± 0.08 defg**	69.62 ± 4.26 c**	20.64 ± 3.02 fg**	2.51 ± 0.2 ef**	14.57 ± 1.84 g**	
C3	1.09 ± 0.09 bcd**	85.17 ± 6.58 b**	23.77 ± 2.45 ef**	2.54 ± 0.18 ef**	17.66 ± 2.01 defg**	
D1	1.18 ± 0.09 b**	82.96 ± 8.62 b**	58.07 ± 3.99 a**	2.32 ± 0.23 efg**	21.5 ± 1.51 bcd**	
D2	1.44 ± 0.08 a**	113.49 ± 8.36 a**	42.37 ± 4.23 b**	2.17 ± 0.24 fg**	17.48 ± 1.68 defg**	
D3	1.37 ± 0.11 a**	109.23 ± 7.69 a**	53.6 ± 4.56 a**	3.49 ± 0.21 cd**	16.63 ± 1.75 efg**	
CK1	1.21 ± 0.1 b**	70.03 ± 2.41 c**	31.12 ± 4.75 d**	2.09 ± 0.22 fgh**	29.27 ± 2.36 a**	
CK2	1.14 ± 0.09 bc**	53.66 ± 2.33 d**	27.51 ± 3.25 de**	1.62 ± 0.21 hi**	17.19 ± 1.44 efg**	
CK3	1.01 ± 0.11 c**	34.38 ± 2.57 fg**	17.93 ± 2.06 g**	1.55 ± 0.2 i**	15.98 ± 1.51 fg**	
CK4	0.93 ± 0.1 bc**	26.7 ± 2.48 g**	12.03 ± 1.09 h**	1.58 ± 0.18 i**	17.85 ± 4.66 defg**	

A, B, C, and D represent common organic fertilizer, bio-organic fertilizer, sheep manure and rapeseed cake fertilizer, respectively. Numbers 1, 2, and 3 after A, B, C, and D for application concentrations of 10 g/kg (1%), 50 g/kg (5%) and 100 g/kg (10%), respectively. CK1: monthly applications of 25 g compound fertilizer, CK2: monthly applications of 25 g urea, CK3: only 5 ‰ urea twice per month, CK4: no fertilizers. Lowercase letters after numbers in the same column represent the difference was significant at *p* < 0.05 level. * and ** represent the difference is significant at *p* < 0.05 level and *p* < 0.01 levels, respectively.

## Data Availability

The data presented in this study are available on request from the corresponding author.
